# Germline variants associated with leukocyte genes predict tumor recurrence in breast cancer patients

**DOI:** 10.1038/s41698-019-0100-7

**Published:** 2019-11-01

**Authors:** Jean-Sébastien Milanese, Chabane Tibiche, Jinfeng Zou, Zhigang Meng, Andre Nantel, Simon Drouin, Richard Marcotte, Edwin Wang

**Affiliations:** 1 National Research Council Canada, 6100 Royalmount Avenue, Montreal, QC H4P 2R2 Canada; 2 Department of Biochemistry & Molecular Biology, Medical Genetics, and Oncology, University of Calgary, 3330 Hospital Drive NW, Calgary, AB T2N 4N1 Canada; 3 Chinese Academy of Agricultural Science, No. 12 Zhongguangcun South Street, Haidian District, Beijing, 100086 China; 4 Rosalind and Morris Goodman Cancer Research Centre, McGill University, 1160 Pine Avenue W, Montreal, QC H3A 1A3 Canada; 5 Alberta Children’s Hospital Research Institute and Arnie Charbonneau Cancer Research Institute, University of Calgary, 3330 Hospital Drive NW, Calgary, AB T2N 4N1 Canada

**Keywords:** Cancer models, Cancer genetics, Predictive markers, Computational biology and bioinformatics

## Abstract

Germline variants such as BRCA1/2 play an important role in tumorigenesis and clinical outcomes of cancer patients. However, only a small fraction (i.e., 5–10%) of inherited variants has been associated with clinical outcomes (e.g., BRCA1/2, APC, TP53, PTEN and so on). The challenge remains in using these inherited germline variants to predict clinical outcomes of cancer patient population. In an attempt to solve this issue, we applied our recently developed algorithm, eTumorMetastasis, which constructs predictive models, on exome sequencing data to ER+ breast (*n* = 755) cancer patients. Gene signatures derived from the genes containing functionally germline variants significantly distinguished recurred and non-recurred patients in two ER+ breast cancer independent cohorts (*n* = 200 and 295, *P* = 1.4 × 10^−3^). Furthermore, we compared our results with the widely known Oncotype DX test (i.e., Oncotype DX breast cancer recurrence score) and outperformed prediction for both high- and low-risk groups. Finally, we found that recurred patients possessed a higher rate of germline variants. In addition, the inherited germline variants from these gene signatures were predominately enriched in T cell function, antigen presentation, and cytokine interactions, likely impairing the adaptive and innate immune response thus favoring a pro-tumorigenic environment. Hence, germline genomic information could be used for developing non-invasive genomic tests for predicting patients’ outcomes in breast cancer.

## Introduction

Cancer is a process of asexual evolution driven by genomic alterations. A single normal cell randomly acquires a series of mutations that allows it to proliferate and to be transformed into a cancer cell (i.e., founding clone), which initiates tumor progression and recurrence. In general, cancer recurrence and metastasis are the result of the interactions of multiple mutated genes. New somatic mutations arise and are selected if they confer a selective fitness advantage (e.g., proliferation, survival, etc.) to a founding clone in the context of a pre-existing genomic landscape (i.e., germline variants). Hence, pre-existing germline variants provide a profound constraint on the evolution of tumor founding clones and subclones and therefore have a contingent effect on the genetic makeup of tumor and presumably patient outcomes. Family history remains one of the major risk factors that contribute to cancer, and recent studies have identified several genes whose germline mutations are associated with cancer. For example, patients suffering from Li–Fraumeni syndrome have an almost 100% chance of developing a wide range of malignancies before the age of 70 years. Most patients carry a missing or damaged *p53* gene, a tumor suppressor whose activity is impaired in almost 50% of all cancers. Other cancer-predisposition genes include *BRCA1* and *BRCA2*,^1,2^ which are associated with breast and ovarian cancer; *PTEN*,^3^ whose mutation results in Cowden syndrome; *APC*, which is linked to familial adenomatous polyposis;^4^ and the Retinoblastoma gene *RB1*.^5^ Two distinct types of multiple endocrine neoplasias are associated with the *RET* and *MEN1*^6^ genes while *VHL* alterations result in kidney and other types of cancer.^7^ Finally, Lynch syndrome, a form of colorectal cancer, is linked to *MSH2*, *MLH1*, *MSH6*, *PMS2*, and *EPCAM*.^8^ Genetic tests based on these highly penetrant gene mutations have shown their usefulness, but they can explain only a small fraction (5–10%) of patients. When neoplasms arise, they are modulated by the interactions of multiple genes based on a great diversity of genetic alterations, which leads to high tumoral heterogeneity.

Thus far, it is unclear to what extent germline variants affect tumorigenesis. We have previously shown that tumor founding clone mutations are able to predict tumor recurrence.^9^ Here we reasoned that the collective impact of germline variants in cancer patients might largely determine tumorigenesis, evolution, and even clinical outcomes. That is, germline variants act in combination with newly acquired somatic mutations to modulate tumorigenesis and tumor recurrence. The combination of germline variants and somatic mutations of each patient predispose specific activation of biological/signaling pathways (even phenotypes) that directly impact clinical outcomes. Therefore, the germline genomic landscape of cancer patients might predict disease progression. Yet, clinical outcome predictions using cancer germline genomic information have been limited to only a few cancer types or to a limited number of genes.^1–8^ The increasing availability of genome sequencing data provide opportunities to develop predictive models that can translate these complex genomic alterations into clinical use.

Breast cancer patients with no lymph node involvement often undergo unnecessary adjuvant chemotherapy treatment (70–80% of patients). In fact, toxic therapies are given to most women with early-stage breast cancer from which 60–75% will not receive any benefit but instead will experience only side effects.^9^ Therefore, biomarkers’ identification to accurately stratify low-risk breast cancer patients who will not benefit from adjuvant chemotherapy is essential. The ITRANSBIG Consortium suggests that, to be clinically practicable, low-risk patients should be associated with 10-year overall survival probabilities of at least 88% for ER+ tumors. Prognostic biomarkers, such as ours, can predict whether a patient is more likely to suffer from tumor recurrence, which would aid greatly clinicians in making treatment decisions.

In this study, we showed that the collective germline variants of breast cancer patients predict tumor recurrence by applying a recently developed method, eTumorMetastasis,^10^ to 755 breast cancer patients. In addition, we showed that these results also outperformed the most popular prognostic test Oncotype DX.^11,12^ Further statistical analyses showed that the leukocyte gene expression levels and tumor-infiltrating leukocytes (TILs) fractions within tumors between the two predicted groups were significantly different. Germline variants associated with tumor recurrence likely impair the adaptive immune response functions of affected individuals, increasing the susceptibility to relapse. These results highlight the important role of germline variants in tumor evolution and recurrence.

## Results

### Germline variants predict breast cancer recurrence

To examine whether germline variants were able to predict tumor recurrence, we used whole-exome sequencing data (i.e., from the National Cancer Institute (NCI) Genomic Data Commons (GDC)) of healthy tissues from 755 estrogen receptor-positive (ER+) breast patients by applying our recently developed method, eTumorMetastasis.^10^ ER+ subtype represents ~70% of breast cancer patients, thus, in this study, we used only patient data from this subtype. The demographic table of the breast cancer cohort is represented in Table 1.

**Table 1:** Demographic and clinical characteristics for ER+ breast cancer samples

Variable	Training set (*n* = 200)		Validation set 1, TCGA-CPTAC (*n* = 295)		Validation set 2, TCGA Nature (*n* = 200)	
Clinical characteristic	Number of patients	Percentage	Number of patients	Percentage	Number of patients	Percentage
Age, years						
Median	59		60		58	
≤59	102	51	149	50.5	105	52.5
>59	98	49	146	49.5	95	47.5
Death						
Yes	29	14.5	33	11.2	26	13
No	171	85.5	262	88.8	174	87
Stage						
I	37	18.5	53	17.9	30	15
II	108	54	164	55.6	113	56.5
III	40	20	72	24.4	49	24.5
IV	8	4	2	0.7	4	2
X	5	2.5	2	0.7	3	1.5
NA	2	1	2	0.7	1	0.5
Subtype						
Luminal A	95	47.5	38	12.9	58	29
Luminal B	42	21	18	6.1	46	23
Unknown	10	5	11	3.7	21	10.5
NA	53	26.5	228	77.3	75	37.5
Nodal status						
0	87	43.5	129	43.7	87	43.5
1–2	102	51	130	44.1	92	46
3	7	3.5	30	10.2	18	9
X	4	2	6	2	3	1.5
Relapse						
Yes	30	15	34	11.5	20	10
No	170	85	261	88.5	180	90
DFS, months						
Median	49.3		32		34.2	
≤38.5	97	48.5	197	66.8	126	63
>38.5	86	43	73	24.7	56	28
NA	17	8.5	25	8.5	18	9

**Fig. 1:**
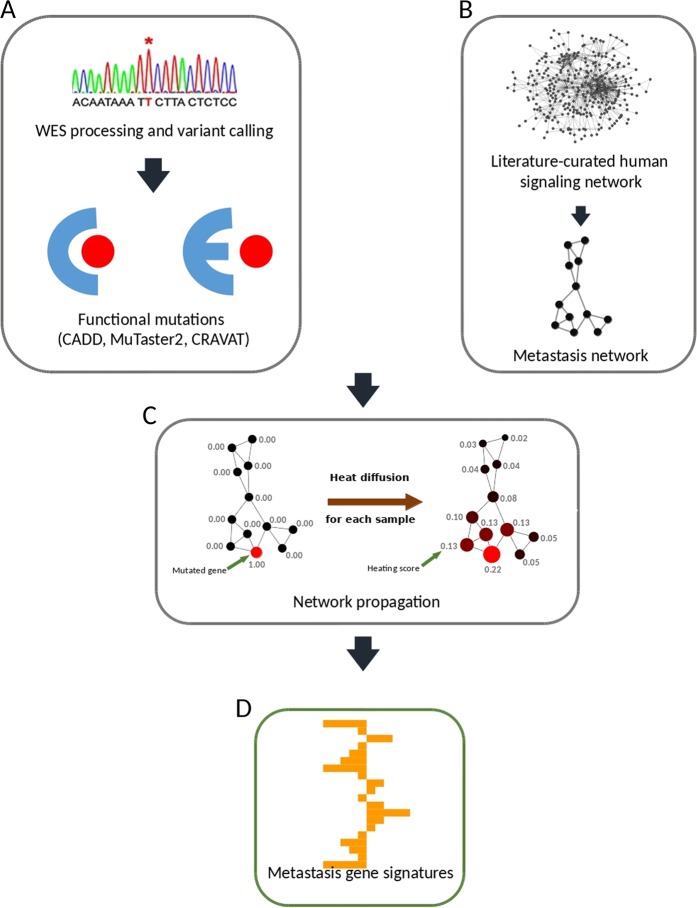
A flowchart of eTumorMetastasis. **a** Germline variants were identified using whole-exome sequencing data of tumors and their paired normal samples. Functional annotation of all variants was performed and non-functional variants were filtered. **b** In parallel, a cancer-specific recurrence network was constructed. **c** Then we used network propagation (or heat diffusion) using the functionally mutated genes as seeds. Seeds act as heating sources and their heat is diffused across the network. Finally, when diffusion is complete, a “heating score” is assigned to each gene. **d** The “heating scores” for all network genes from all samples were then aggregated into a matrix from which we extract NOG signatures

We hypothesized that somatic mutations are evolutionary selected to work with the pre-existing germline variants to initiate tumorigenesis and recurrence. This is the underlying concept of eTumorMetastasis. In turn, the model infers that pre-existing germline variants of cancer patients have predictive power for recurrence and clinical outcomes. eTumorMetastasis contains three main components: (1) a network-based approach^13,14^ to transform functionally genetic variants’ information on a cancer type-specific signaling network; (2) identifying biomarkers via our previously developed method, MSS (Multiple Survival Screening);^15^ and (3) a better predictive power using our previously developed method by combining biomarkers.^16^ The detailed procedure of eTumorMetastasis and network construction were described previously.^10^ A flowchart of the algorithm can be found in Fig. 1. Briefly, we constructed an ER+ breast cancer-specific recurrence signaling network. Then, using germline whole-exome sequencing data of each breast cancer patient, we annotated the germline variants and retained functional genes only (i.e., genes with at least one functional variant). Next, we mapped the functional genes on the recurrence signaling network and conducted network propagation where functional genes act as “heating source”. Network propagation can be described as heat diffusion. The functional genes diffuse their heat across the network allowing us to transform mutation binary data (0s and 1s) into the continuous form. In other words, network propagation enables us to measure the impact of a functional mutation onto a specific context (i.e., recurrence). The second component of eTumorMetastasis is the MSS algorithm, which randomizes genes and samples to provide robust biomarkers (or gene signatures). Finally, the third component consists of an ensemble-based approach combining multiple biomarkers to improve prediction accuracy (see “Methods” and Supplementary Methods).

**Fig. 2:**
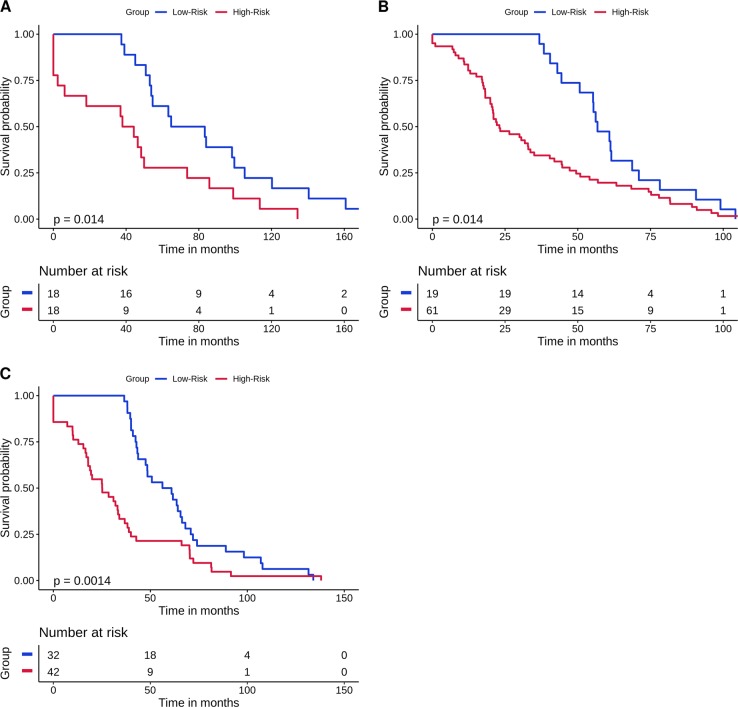
Kaplan–Meier curves of the risk groups for breast cancer patients predicted by the NOG_CSS sets. Samples without DFS time or who could not be predicted were removed. NOG_CSS sets derived from germline mutations in **a** the training set, **b** the validation set, TCGA-Nature, and **c** the validation set, TCGA-CPTAC. Blue and red curves represent low- and high-risk groups, respectively. *P* values were obtained from two-sided *χ*^2^ test

We used the germline genomic information of 200 ER+ breast cancer samples (i.e., training samples) to identify gene signatures (i.e., because eTumorMetastasis identifies network-based gene signatures, we called the gene signatures Network Operational Signatures or NOG signatures), which could distinguish recurred and non-recurred breast tumors. By applying eTumorMetastasis to the germline genomes of 200 patients, we identified 18 NOG signatures (Tables S1 and S2) for ER+ breast cancer. Each NOG contains 30 genes and represents a cancer hallmark such as apoptosis, cell proliferation, cell cycle, and so on. We have previously shown that multiple gene signatures representing distinct cancer hallmarks could be identified from one training cohort.^15^ Furthermore, ensemble-based prediction using multiple gene signatures representing distinct cancer hallmarks significantly improved prediction performance.^16^ Thus we used all 18 NOG gene signatures to construct a NOG_CSS (i.e., NOG-based Combinatory Signature Set) by applying it to a testing set of 60 samples (Table S3) similar to the method we previously developed.^16^ Finally, based on the NOG_CSS, we successfully predicted the prognosis of ER+ breast cancer patients. As shown in Fig. 2 and Table 2, we demonstrated that the germline-derived NOG_CSS significantly distinguished recurred and non-recurred breast tumors in two validations sets: 200 (ER+ Nature-Set, *P* = 1.4 × 10^−2^) and 295 (ER+ TCGA-CPTAC independent set, *P* = 1.4 × 10^−3^). These results suggest that germline variants are significantly correlated with tumor recurrence and support our hypothesis that the original germline genomic landscape of a cancer patient has a significant impact on clinical outcome.

**Table 2:** Prediction accuracy and recall rate for validation sets for breast cancer using the NOG_CSS sets derived from germline mutations

Dataset	Number of samples	Low risk		High risk	
		Accuracy (%)^a^	Recall (%)^b^	Accuracy (%)^c^	Recall (%)^d^
Training set	200	93.8	26.5	27.5	36.7
TCGA-Nature	200	94.9	31.1	8.2	25.0
TCGA-CPTAC	295	93.5	38.7	16.6	20.6

^a^Percentage of non-recurred (i.e., non-metastatic) samples in the predicted low-risk group

^b^Percentage of the predicted low-risk samples from the non-recurred group

^c^Percentage of recurred (i.e., metastatic) samples in the predicted high-risk group

^d^Percentage of the predicted high-risk samples from the recurred group

As a proof of concept and to further demonstrate the constraint given by germline variants onto the tumor development, we used the NOG_CSS and the gene expression of normal tissue of 72 breast cancer patients to predict patients’ relapse risk (see “Methods” for details). Samples were assigned in the training or validation sets previously defined. The results of this prediction can be found in Table 3. Accuracy for low-risk samples was similar to germline variants predictions (88.9% compared to 94.9%), suggesting that the impact from germline variants is also reflected in gene expression and correlates with our hypothesis that gene expression and tumor development are affected directly from germline predispositions. Strikingly, the accuracy obtained for high-risk samples with gene expression data was much better than what we obtained using germline variants (66.7% compared to 21.0%), suggesting that gene expression is a better predictor of recurrence for high-risk patients or that high-risk patients might possess a more complex somatic landscape not captured solely by germline mutations. In addition, we also compared germline variants’ prediction with Oncotype DX breast cancer recurrence score (RS; Table 4) and outperformed accuracies and recalls for both predicted groups (high and low risk; see “Methods” and Supplementary Methods).

**Table 3:** Prediction accuracy and recall rate for validation samples for breast cancer using the NOG_CSS sets derived from gene expression of normal tissue

Dataset	Number of samples	Low risk		High risk	
		Accuracy (%)^a^	Recall (%)^b^	Accuracy (%)^c^	Recall (%)^d^
TCGA-Validation	49	88.9	48.5	66.7	62.5

^a^Percentage of non-recurred (i.e., non-metastatic) samples in the predicted low-risk group

^b^Percentage of the predicted low-risk samples from the non-recurred group

^c^Percentage of recurred (i.e., metastatic) samples in the predicted high-risk group

^d^Percentage of the predicted high-risk samples from the recurred group

**Table 4:** Prediction accuracy and recall rate for breast cancer using Oncotype DX formula and RNA-seq data

Dataset	Number of samples	Low risk		High risk	
		Precision (%)^a^	Recall (%)^b^	Precision (%)^c^	Recall (%)^d^
Training Set	200	84.8	16.6	18.8	40.0
TCGA-Nature	200	90.0	20.0	6.5	20.0
TCGA-CPTAC	295	86.0	16.6	10.1	26.5

^a^Percentage of non-recurred (i.e., non-metastatic) samples in the predicted low-risk group

^b^Percentage of the predicted low-risk samples from the non-recurred group

^c^Percentage of recurred (i.e., metastatic) samples in the predicted high-risk group

^d^Percentage of the predicted high-risk samples from the recurred group

To compare the prediction performance of the NOG_CSS with clinical factors, we conducted relapse-free survival analysis of clinical factors using Cox proportional hazards regression model. The best *P* value (i.e., *P* = 2.0 × 10^−2^, log-rank test) using covariate models (Table S4) was not better than the one derived from the germline NOG_CSS (*P* = 1.4 × 10^−3^). These results suggest that gene signatures derived from germline genomic information have a better predictive performance than clinical factors.

**Fig. 3:**
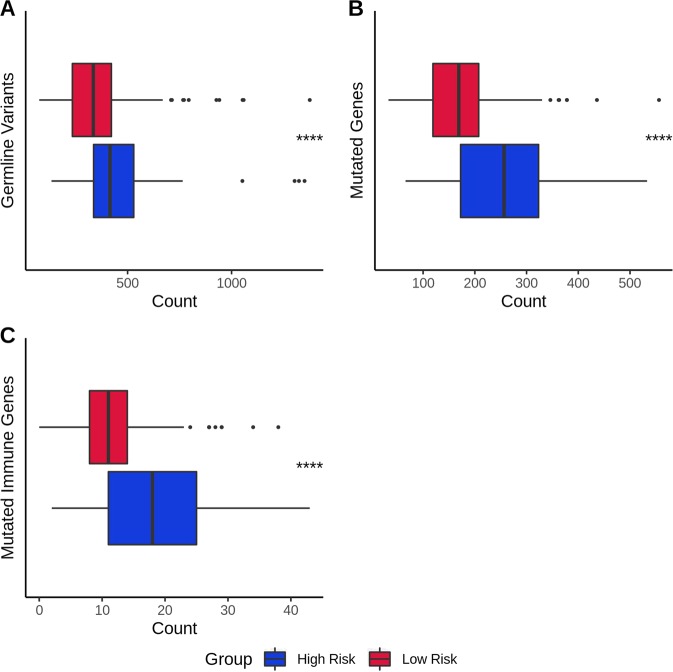
Boxplot comparison of functional germline variants and genes for the predicted risk groups. Samples who could not be predicted were removed. **a** Functional germline variants. **b** Functionally mutated genes. **c** Functional germline mutated immune genes. *P* values were obtained from two-sided Student’s *t* test. *P* value significance: ****<0.0001. Outliers are shown as individual points

Finally, we also assessed the number of functional germline variants in all genes or genes specifically expressed in leukocytes as well as the number of genes harboring germline variants for both the predicted risk group. Two-sided Student’s *t* tests revealed a significant difference for all the comparisons (1.29 × 10^−13^, 8.24 × 10^−16^, and 1.14 × 10^−5^, respectively), with functional germline variants in leukocyte-expressed genes being the most indicative distinction. All distributions are highlighted in Fig. 3. A higher germline functional mutation count for high-risk group suggests once again that germline variants have a significant impact on tumor development and therefore recurrence.

### Predictive germline variants could impair the immune system

**Fig. 4:**
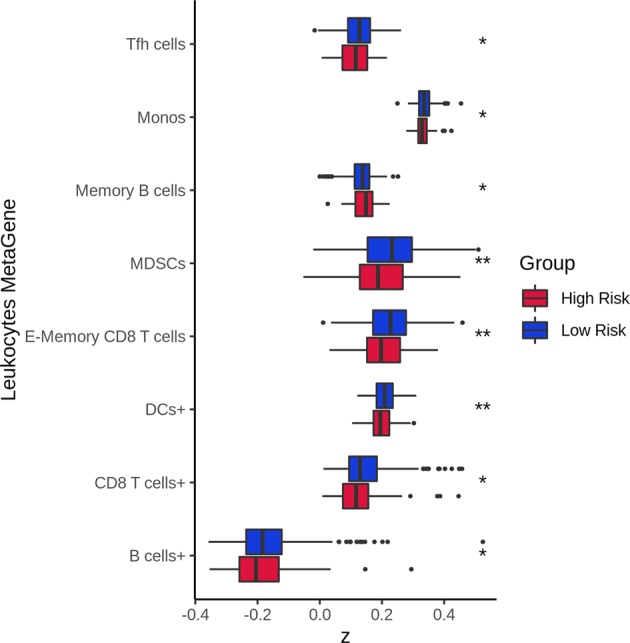
Boxplot comparison of leukocyte expression profiles for the predicted risk groups. Samples who could not be predicted were removed. For a complete analysis, see Fig. S1. *P* values were obtained from two-sided Student’s *t* test. *P* value significance: *<0.05, **<0.01. Outliers are shown as individual points

**Fig. 5:**
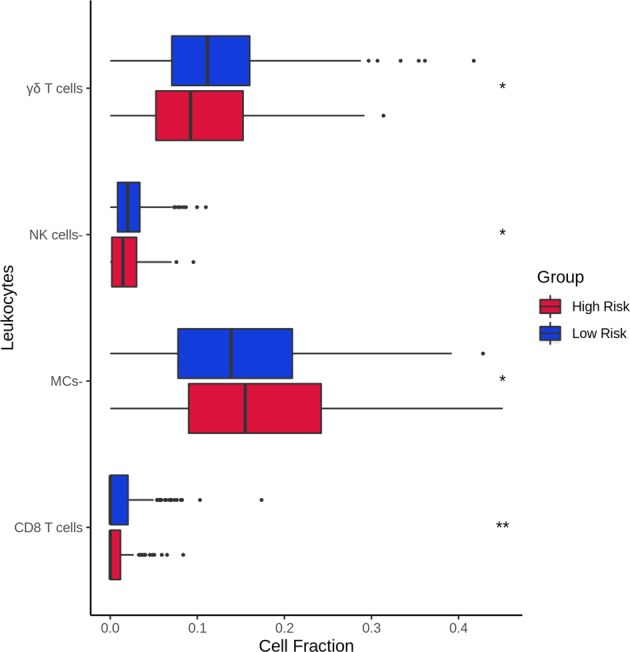
Boxplot comparison of leukocyte cell fractions for the predicted risk groups. Samples who could not be predicted were removed. For a complete analysis, see Fig. S2. *P* values were obtained from two-sided Student’s *t* test. *P* value significance: *<0.05, **<0.01. Outliers are shown as individual points

To further understand why germline genomic landscapes of cancer patients are predictive for tumor recurrence, we ran enrichment analyses for genes present in the NOG signatures of breast cancers using DAVID.^17^ Interestingly, most genes were enriched in immune- or cell proliferation-related biological pathways and Gene Ontology terms (Table S5). Thus we hypothesized that recurred patients have more functionally inherited variants in immune system-related genes than non-recurred patients. To test this hypothesis, we compared gene expression for leukocyte metagenes between predicted recurred and non-recurred patients from tumor transcriptomes. The leukocyte metagene list was obtained from a recent study.^18^ Two-sided Student’s *t* tests between both groups revealed a significant difference for myeloid-derived suppressor cells (MDSCs), effector memory CD8 T cells (E-Memory CD8+ T cells), activated dendritic cells (DC cells+), activated CD8 T cells (CD8+ T cells), T follicular helper cells (Tfh), monocytes (Monos), memory B cells, and activated B cells (B cell+; *P* = 1.99 × 10^−3^, *P* = 4.03 × 10^−3^, *P* = 6.67 × 10^−3^, *P* = 2.10 × 10^−2^, *P* = 2.30 × 10^−2^, *P* = 3.78 × 10^−2^, *P* = 4.37 × 10^−2^, and *P* = 4.46 × 10^−2^, respectively). To a similar extent, we also analyzed TILs’ fractions to see whether these were different between the predicted groups (CIBERSORT LM22, see “Methods”).^18,19^ Two-sided Student’s *t* tests revealed a significant difference in TILs’ fractions for gamma delta T cells (γδ T cells), resting natural killer cells (NK cells−), resting mast cells (MCs−), and CD8+ T cells (*P* = 3.14 × 10^−2^, *P* = 4.29 × 10^−2^, *P* = 4.97 × 10^−2^, *P* = 8.21 × 10^−3^, respectively). A better representation of leukocyte gene expression profiles and TILs’ fractions between the predicted groups are shown in Figs 4 and 5, respectively, and the complete abbreviation lists can be found in Tables S6 and S7. Overall, these results suggest that germline variants of cancer patients could directly influence gene expression and alter immune system functions, cell division, and the immune tumor microenvironment (TME). Modulation of these pathways would then affect recurrence and patient outcome.

To further investigate the predictive power of variants in leukocyte-expressed genes, we re-ran eTumorMetastasis^10^ pipeline using only functional germline variants in leukocyte-expressed genes. Interestingly, we were not able to obtain enough germline variants in leukocyte-expressed genes as network seeds in each sample to extract a gene signature proposing leukocyte variants only provides partial information and the complete germline mutational landscape is more representative (more details in Supplementary Methods).

## Discussion

We developed a risk classification method using germline genomic variants to predict clinical outcomes and demonstrated that these germline variants shape tumor evolution and recurrence. The enrichment analysis of the NOG signatures derived from germline variants suggest that recurred patients differently regulate signaling pathways associated with immune responses (such as inflammation and cell adhesion). Comparison with Oncotype DX suggests that germline variants could also predict tumor recurrence (94.9% versus 90.0%, Tables 2 and 4). Comparison of germline variants and affected genes between the two predicted groups indicates that these variants are predisposing to cancer. A significantly higher number of functional variants could lead to a greater number of impaired proteins that would create an imbalance in signaling pathways, favoring tumor development and recurrence. Moreover, we found that leukocyte genes harbored a greater number of germline variants in the predicted high-risk group. These germline variants likely impede the immune system, leading to a more favorable environment for tumor development.

We found that germline variants in genes regulating cell division, immune cell infiltration, and T cell activities are predominately predictive for tumor recurrence. More specifically, mutations in the antigen processing and presentation pathway could impair neoantigen presentation at the surface of cancer cells so that T cells are no longer able to recognize tumor cells, allowing them to evade immune detection. Furthermore, mutations in cell division process could introduce a higher number of somatic mutations during cell division directly promoting tumor development. Activation of Wnt pathway can also block the infiltration of immune cells within tumors.^20^ TILs’ expression analysis also reveal strong correlation with germline prediction and differential expression in MDSCs, CD8+ T cells, DCs, Tfh cells, monocytes, and B cells (Fig. 4). Aside from memory B cells, all other TILs were enriched in the predicted low-risk group. B cells have been shown to secrete pro-tumorigenic factors (e.g., angiogenesis, tumor growth) and also to inhibit the antitumor immune response via cytokines.^21–23^ DCs are well known for their role in antigen presentations and in initiating an adaptive immune response.^24^ Tfh cells have been shown to favor an adaptive immune response via the B cell chemoattractant CXLC13 in breast cancer.^25^ Along with E-memory CD4 T cells, E-memory CD8 T cells possess a key role in the immune response and tumor infiltration. Patient survival has been directly correlated with CD8 T cells infiltration. Multiple mechanisms are used by cancer cells to escape immune responses such as altering cytokine and chemokine attraction to create a non-inflammatory environment, which, in turn, inhibits T cell infiltration.^26,27^ Monocytes and MDSCs have largely been associated with tumor recurrence in the literature. Monocyte differentiation into tumor-associated macrophages promotes anti-immunity signals such as angiogenesis and growth factors resulting in a TME favoring cancer cell proliferation. However, there have been some reports indicating that a nonclassical monocyte subtype, patrolling monocytes, reduces tumor recurrence by recruiting NK cells.^28,29^ Monocytes can also differentiate into pro-inflammatory M1 macrophages aiding the adaptive immune response. A recent study has also shown that tumor necrosis factor-α (TNFα) secreted by T cells induces emergency myelopoiesis resulting in an increase in MDSCs in mice.^30^ TNFα secretion by T cells could be a regulation mechanism induced by the adaptive immune response once a certain concentration of T cells has infiltrated the tumor. This point could explain the higher expression numbers for MDSCs in predicted low-risk samples.

A significant difference was also seen in TILs’ cell fractions of γδ T cells, CD8 T cells, NK cells−, and MCs (Fig. 5) between both the predicted groups. CD8 T cell tumor infiltration is crucial for an optimal immune response; these cells were present in greater numbers in the predicted low-risk group. γδ T cells are known to have dual effects, capable of exerting both pro-tumor or antitumor response depending on their subtype.^31^ γδT1, γδT-APC, and γδTfh subtypes all possess antitumor activities such as secreting chemoattracting chemokines (i.e., CXLC13), antigen presentation, and antibody-dependent cell-mediated cytotoxicity toward cancer cells.^32^ In breast cancer, MCs are linked with pro-angiogenic factors such as inflammation^33,34^ reflecting a higher MC count in the predicted high-risk group. Finally, NK cells have cytotoxic abilities and a greater number in tumors is indicative of a good prognosis.^35,36^

Our understanding of the biology mediating recurrence is limited. Germline variants of cancer patients could affect the activity of the immune system in TMEs. For example, germline-encoded receptor variants were shown to trigger innate immune response in cancer patients.^37^ In addition, lung cancer patients with a germline mutation in Nrf2 have a good prognosis because these variants regulate the inflammatory status and redox balance of the hematopoietic and immune systems of cancer patients.^38^ In prostate cancer, patients with a germline variant of the ASPN D locus are associated with poorer outcomes.^39^ These studies, including our own, highlight the impacts of germline variants on tumor recurrence and provide a rationale to further study the effect of germline genomic landscapes on clinical outcomes of carcinogenesis.

Good accuracy obtained using normal tissue RNA prediction shows that germline variants directly influence gene expression and, consequently, tumor development. A higher accuracy for the high-risk group also highlights that gene expression holds a better predictive power than genome sequencing. These results are not surprising considering that gene expression integrates more information than gene-coding mutations alone (e.g., gene regulation). Even the most damaging functional mutation in a gene not expressed would have no impact on the phenotype. We also note that this analysis suffers from a small sample size and should be further explored in the future. This also highlights some limitations of the algorithm as genome sequencing is not always the most informative data type. Furthermore, the model relies on a large quantity of samples as input for the NOG signatures to be robust. Finally, good clinical metadata for each sample is also crucial to allow a clear disparity between the groups of interest.

In all, these results suggest that germline variants modulate the immune system and the immune TME, which in turn stimulate tumor recurrence and ultimately affect patient outcome. Traditionally, germline variants have been largely ignored in the cancer genomic community; for example, most of the cancer genomic studies including the GDC and The Cancer Genome Atlas (TCGA) have often focused only on somatic mutations while germline mutations were filtered out before formal analysis of tumor genome sequencing data. The demonstration that germline exome sequencing data can predict cancer patients’ outcomes suggests that non-invasive genomic tests of cancer patients could be devised to determine cancer prognosis and inform treatment decisions. Genome-wide germline variants can be easily identified by genome/whole-exome sequencing of liquid biopsies such as blood or saliva samples. Prognostic prediction using a patient’s germline genomic landscape opens up the possibility of assessing cancer patients’ risk of recurrence, which allows for a better forecasting of cancer recurrence in a quick, convenient, and non-invasive manner. Germline genomic testing could provide cheaper alternatives to current prognostic tests used in clinical environment such as Oncotype DX.

## Methods

### Exome data processing

We obtained whole-exome sequencing data of breast cancers from the NCI GDC. We collected 755 ER+ breast cancer samples: a training set of 200 samples, a testing set of 60 samples, and two independent validation sets of 200 and 295 samples (TCGA-Nature and TCGA-CPTAC, respectively, Table S8). Raw sequence reads from healthy samples of cancer patients were processed in compliance with GATK^40^ best practices pre-processing pipeline and the method described previously.^10^ Variant calling was then performed using Varscan2.^41^ Patient consent was obtained through the NCI GDC policies in compliance with the Health Insurance Portability and Accountability Act guidelines. The ethics of this study have been approved by the National Research Council of Canada.

### Transcriptome data processing

Normal tissue RNA-seq is less accessible on the GDC than tumor RNA-seq data. Out of the 755 samples in our dataset, we were only able to find 72 samples from which normal tissue RNA-seq was available. FPKM (fragments per kilobase of transcript per million mapped reads) values for each sample were downloaded and then normalized using *z*-score normalization. Each sample was then assigned to our previously defined training and validation set (23 and 49, respectively).

### Germline variant identification

To determine germline variants, we used variant allele frequencies (VAFs) between the tumor and healthy samples. We defined homozygous germline variants if the VAF in the healthy samples was ≥90. For heterozygous germline variants, we used the VAF cutoffs between 45% and 65% in normal samples. Functional annotation was performed using CADD,^42^ MutationTaster,^43^ and CRAVAT.^44^ Only germline functional variants were retained for downstream analysis.

### Germline NOG signature identification

To identify NOG signatures using the functional mutated genes of breast cancer patients’ germline genomes, we followed the eTumorMetastasis^10^ method (Fig. 1). Briefly, a cancer-specific recurrence network was constructed using gene expression data associated with cancer recurrence combined with a literature-curated signaling network. The final ER+ breast cancer-specific recurrence network contained 6148 genes and 62,004 interactions. For each patient, we used its germline functionally mutated genes as seeds on the network and performed network propagation (similar to heat diffusion). The impact of germline functionally mutated genes can then be applied in a recurrence context (network) and each gene is ultimately assigned a “heating score.” Then we aggregate those scores together and run MSS to extract germline NOG signatures. More details about the network construction, MSS, or each step in the algorithm can be found in our previous publications.^10,15^

### Transcriptomic normal tissue prediction

Like mentioned above, each sample was assigned to our previously defined training and validation set (23 and 49, respectively). Accuracy and recall rate were obtained using a similar approach as with the eTumorMetastasis^10^ method. For all 18 NOG signatures previously identified with genome sequencing, we calculated centroid values for each gene between both groups (high and low risk) in the training set. In this case, centroid values were obtained from gene expression values instead of network propagation scores. We used leave-one-out cross-validation to classify each sample in the validation set. Centroids from both groups were calculated, and based on Pearson correlation, each sample was assigned to its closest group (low, high risk). We built a NOG_CSS using the same cutoffs obtained from genome sequencing. Prediction accuracy and recall rate for validation samples can be found in Table 3.

### Oncotype DX and germline variant comparison

The Oncotype DX breast cancer RS is the most popular genomic test for cancer prognosis. For each patient, it assesses the recurrence risk and benefits from chemotherapy treatment. The test uses the expression values of 21 genes to calculate an RS for ER+ breast cancer patients using a formula (Supplementary Methods).^11,12^ Gene expression values can be obtained from microarray, reverse transcriptase PCR, or RNA-seq.^45^ Based on the RS, a patient will be assigned into low, intermediate, or high risk. As a comparative analysis, we applied the Oncotype DX formula to our dataset using the normalized RNA-seq data downloaded from the GDC (FPKM-UQ, 751 samples in total). Accuracy and recall obtained from Oncotype DX score are shown in Table 4.

### Leukocyte metagene expression and cell fractions

Leukocyte metagene expression profiles derived from tumor RNA-seq data were obtained from The Cancer Immunome Atlas (TCIA)^18^ and were applied *z*-score normalization. In total, scores for 29 leukocyte metagene profiles were downloaded. Leukocyte cell fractions were also downloaded from TCIA for all 755 breast cancer samples. CIBERSORT^19^ signature of 22 leukocytes was used (LM22).

### Reporting summary

Further information on experimental design is available in the Nature Research Reporting Summary linked to this paper.

## Supplementary information

Supplementary Material

Nature research reporting summary form

## Data Availability

The data that support the findings of this study are publicly available on the GDC data portal under the TCGA-BRCA project (https://portal.gdc.cancer.gov/).

## Code availability

The complete pseudocode for the algorithm can be found in our previous publication (10.1101/268680). R scripts for MSS and network propagation as well as examples on how to run the algorithm can be found in our github repository (https://github.com/WangEdwinLab/eTumorMetastasis).
