# Supplementary material for: Germline variants associated with leukocyte genes predict tumor recurrence in breast cancer patients
Source: NPJ Precis Oncol. 2019 Nov 1;3:28. doi: 10.1038/s41698-019-0100-7 (PMC6825127; doi:10.1038/s41698-019-0100-7)
Supplement: Supplementary file 1 — Supplementary Material [file 41698_2019_100_MOESM1_ESM.docx]

**Supplementary Information**

**Germline variants associated with leukocyte-genes predict tumor recurrence in breast cancer patients**

**Supplementary Figures**

**
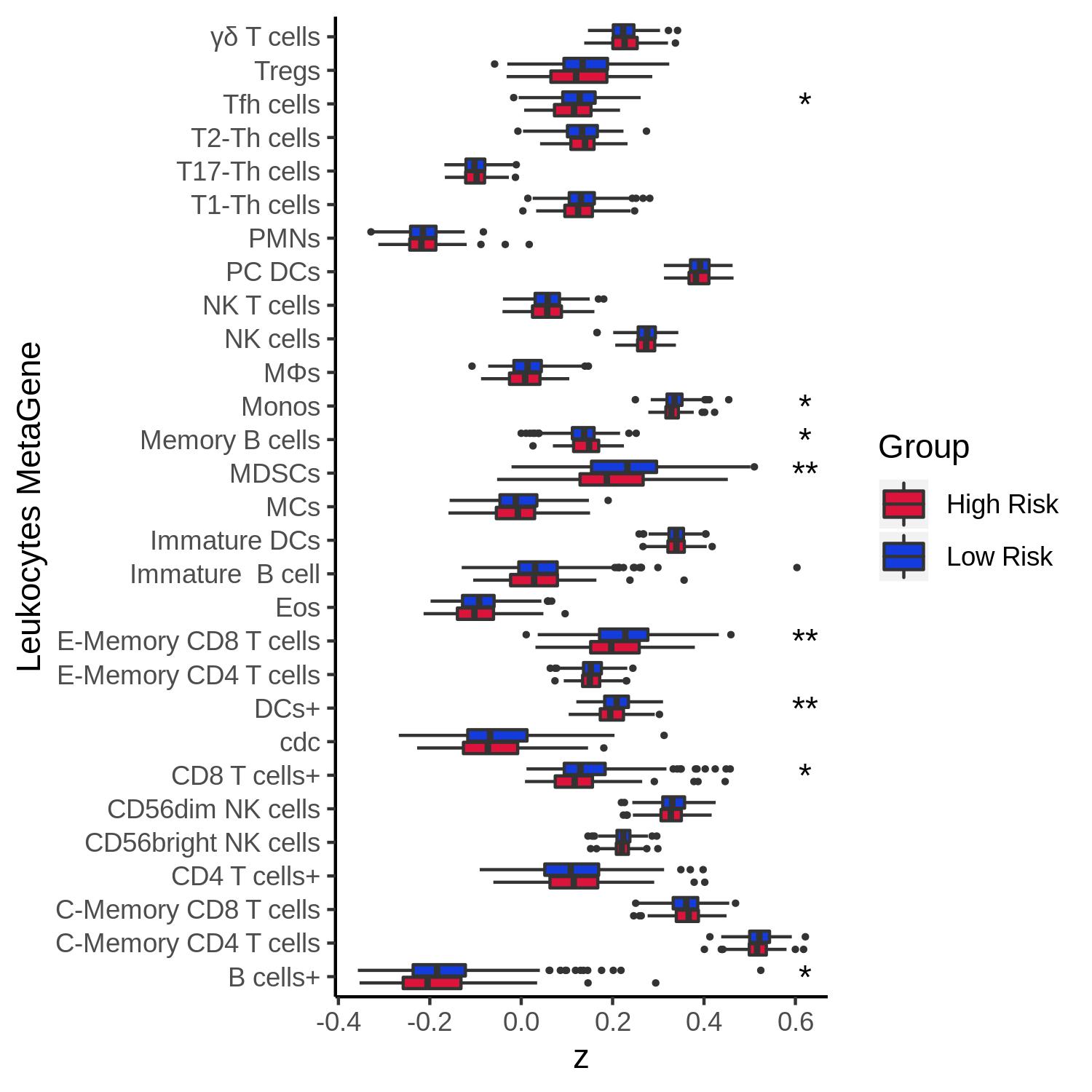
**

**Supplementary Figure 1 Boxplots comparison of leukocyte expression profiles for predicted risk groups. Samples who couldn't be predicted were removed.** P-Values were obtained from Student's t-test. P-Value significance: * < 0.05, ** < 0.01. Outliers are shown as individual points.

**
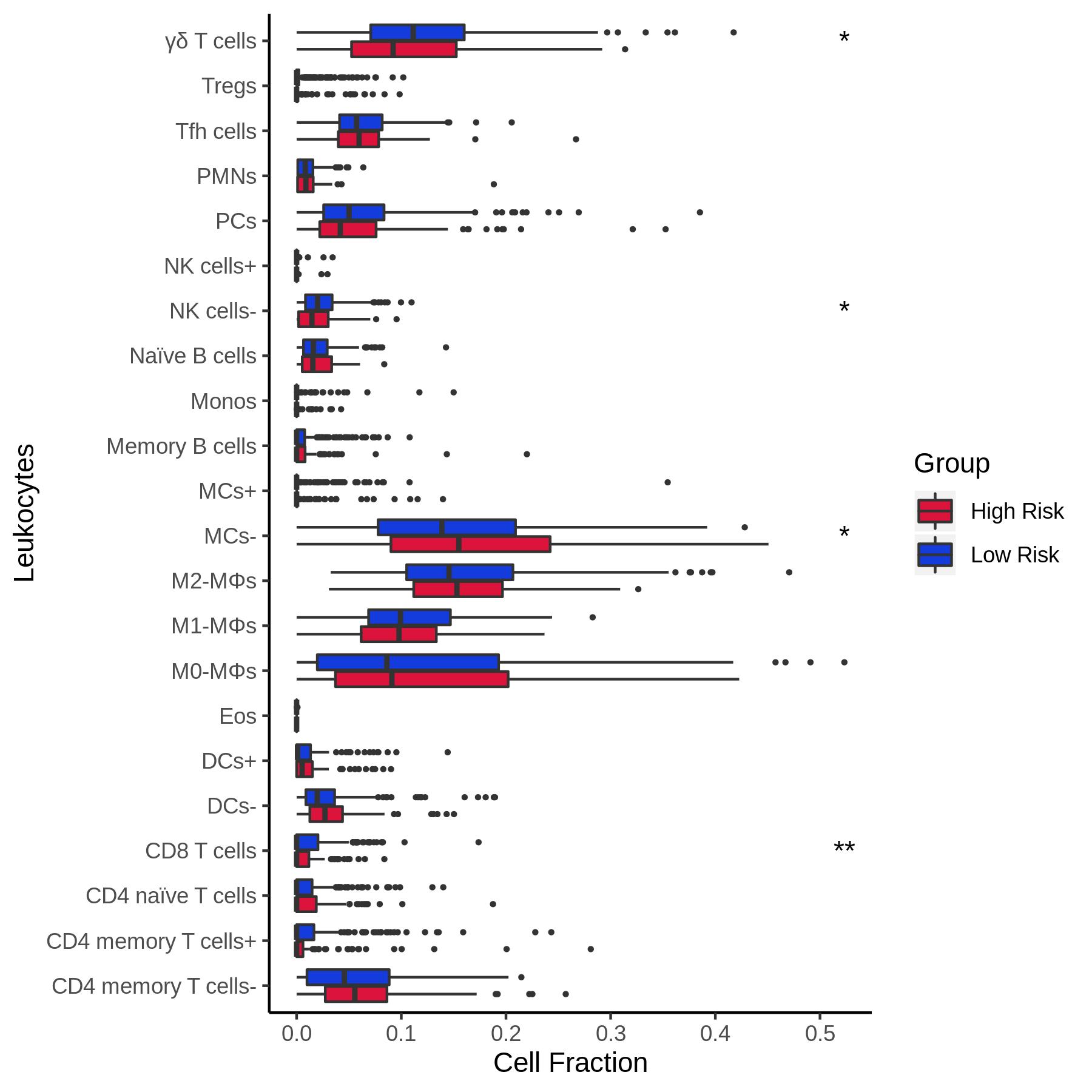
**

**Supplementary Figure 2 Boxplots comparison of leukocyte cell fractions for predicted risk groups. Samples who couldn't be predicted were removed.** P-Values were obtained from Student's t-test. P-Value significance: * < 0.05, ** < 0.01. Outliers are shown as individual points.

**Supplementary Tables**

**Supplementary Table 1 List of genes of network operational signatures derived from breast cancer germline mutations**

| **Apop1** | **Apop2** | **Apop3** | **CCycle1** | **CCycle2** | **Ccycle3** | **Cell Adh1** | **Cell Adh2** | **CellAdh3** | **Cytosk1** | **Cytosk2** | **Cytosk3** | **Imm Res1** | **Imm Res2** | **ImmRes3** | **Prolif1** | **Prolif2** | **Prolif3** |
| --- | --- | --- | --- | --- | --- | --- | --- | --- | --- | --- | --- | --- | --- | --- | --- | --- | --- |
| BRCA1 | APOE | ANXA1 | ACVR1 | BRSK1 | BRCA1 | ADAM17 | ADAM17 | ADAM17 | ACTA2 | ACTRT1 | ACTRT1 | CD19 | BCAP31 | B2M | AGT | ADRA1B | ANXA1 |
| CASP8 | AVEN | BCAP31 | ANXA1 | CCNB1 | C13orf34 | AMBN | BRCA1 | CAV1 | ACTR2 | APOE | AKAP9 | CD4 | CCL4 | C3 | AMBN | BIRC2 | BOK |
| CDK11A | BIRC2 | BFAR | ATR | CCNB3 | CCNB1 | BAI1 | CCL4 | CD209 | ACTRT1 | BBS4 | ALMS1 | CD81 | CCR5 | CCL23 | ASCL1 | BMPR2 | CD81 |
| DOCK1 | BRCA1 | BRCA1 | BRD7 | CDK11A | CCNB3 | CD209 | CD209 | COL11A2 | ASAP1 | CASP8 | CASP8 | CHIA | CD160 | CCL3 | CD81 | BRCA1 | CITED1 |
| EP300 | CDK11A | CDK11A | BRSK1 | CHFR | CDC25B | CD4 | COL6A3 | COL6A3 | BRSK1 | CDK5RAP2 | CYTH2 | CIITA | CD19 | CD19 | CITED1 | CD160 | CITED2 |
| ESPL1 | DAPK1 | CITED2 | CASP8AP2 | CLASP1 | CDCA5 | CD58 | CTGF | CTNND1 | CASP8 | CENPJ | DAPK3 | HCST | CD81 | CD81 | CSF1R | CD81 | CKS1B |
| FGF2 | ESPL1 | ESPL1 | CCNB1 | DCTN2 | CDK11A | CDH17 | DDR1 | EPHA3 | CCNB1 | CEP72 | DIAPH2 | HLA-DMB | CIITA | CIITA | DBH | CDK9 | CTF1 |
| GPX1 | GSN | FAIM3 | CCNB3 | DDIT3 | CHFR | CDH4 | EZR | FAT1 | CDC25B | CEP78 | GSG2 | HLA-DOA | FASLG | ENPP2 | GHRL | CITED1 | DAB2 |
| GSN | GULP1 | FBXO7 | CDK11A | E2F6 | FBXO31 | COL5A1 | F11R | FLOT2 | DAPK3 | CKAP5 | GSN | HLA-DOB | HCST | HCST | GNB1 | DCTN2 | DBH |
| HIPK1 | GZMB | GSN | CENPJ | GSG2 | KIF11 | COL6A3 | FAT1 | FN1 | GSN | CLASP1 | LSP1 | HLA-DPA1 | ICAM1 | ICAM2 | IGSF8 | EDNRA | FAS |
| HYAL1 | GZMH | HIPK1 | CHAF1B | KIF11 | NASP | DPP4 | FLOT2 | IBSP | KIF11 | DAPK1 | MAP2K1 | HLA-DQA2 | ICAM2 | ICAM3 | LTK | ENG | GHRL |
| IKBKB | HIPK1 | IER3 | CHFR | MAPK7 | NCAPD2 | ENG | FN1 | ITGAM | LSP1 | DAPK3 | NCAPG | HLA-DRB3 | ICAM3 | IFITM1 | MDM4 | GHRL | GNB1 |
| IL17A | IKBKB | IKBKB | EP300 | MRE11A | NCAPG | FAT1 | ICAM1 | LAYN | NCAPG | DCTN2 | NF1 | HLA-DRB4 | IFITM1 | IL17A | MMP7 | GNB1 | HOXB4 |
| IL7 | IL17A | IL17A | FBXO31 | NASP | NOTCH1 | FLOT2 | IGFALS | LEF1 | NCAPH | DCTN3 | NPM1 | HLA-DRB5 | IL15 | IL21 | NASP | IL2RA | LTK |
| MAP2K7 | IL7 | IL7 | FOXN2 | NCAPD3 | OFD1 | FN1 | ITGA4 | LIMS1 | NF1 | DIAPH1 | PCM1 | ICAM2 | IL17A | IL7 | NCF1 | INHA | MPL |
| NEFH | LTK | LTBR | KIF11 | NCAPG | PIN1 | ITGAL | ITGAV | MAP2K1 | NPM1 | EZR | PCNT | ICAM3 | IL7 | ITGA4 | PIM2 | MRE11A | PAK7 |
| PIM2 | PLEC | MDM4 | LZTS1 | NOTCH1 | PINX1 | LEF1 | JAM3 | MAP2K2 | NUCB1 | GSN | PDPK1 | ICAM4 | IL9 | ITGAL | PLAU | PLAU | PIN1 |
| PSMD5 | PSME1 | MUC1 | MAP2K1 | OFD1 | PLK1 | LY9 | LEF1 | MLLT4 | OFD1 | GTF2F2 | PFN1 | IFITM1 | ITGAL | KLRC1 | PLK1 | PLK1 | PLAU |
| PSME1 | PSME4 | PCNT | MDC1 | P2RY2 | RAD21 | MAP2K2 | MAP2K2 | MOG | PLK1 | LSP1 | PLEKHG6 | IL17A | KLRD1 | NOTCH1 | PPP1R9B | PPP1R9B | PPP1R9B |
| PSME4 | RASSF5 | PIM2 | MTBP | PINX1 | RBBP8 | MOG | MDC1 | MUC4 | PTPN13 | NCAPG | PLK1 | IL18 | MAP3K14 | PDCD1LG2 | RAP1B | PTEN | SAT1 |
| PTK2B | SEMA3A | PSME1 | NASP | PPP1CA | REC8 | MUC4 | MOG | NFASC | RASSF5 | NF1 | PTK2B | IL2RG | MICB | RAET1G | SAT1 | SAT1 | SGK1 |
| RAD21 | SERPINB9 | PSME4 | NCAPG | RAD50 | RNF8 | MYH6 | MUC4 | NLGN4X | RPA1 | NPM1 | RASSF1 | IL7 | NCF4 | RAET1L | SCRIB | SRA1 | SOX9 |
| SEMA3A | SRA1 | SLK | NOTCH1 | RNF8 | RPA1 | NLGN4X | NLGN4X | NRP1 | S100A9 | PLK1 | RASSF5 | ITGAL | NOTCH1 | SPN | SPHK2 | TACC3 | TCF7L2 |
| SMNDC1 | TGFBR2 | TGFB2 | OFD1 | RPA1 | RPS6KA1 | NPNT | NRP1 | OMD | SCNN1A | PSMD10 | RPA1 | NOTCH1 | RAET1E | TLR10 | TCF7L2 | TCF7L2 | TGFB2 |
| TGFBR2 | TNFRSF10B | TGFBR2 | PIM2 | RPS6KA1 | SMC1A | NRP1 | OMD | PAK1 | SORBS1 | RASSF5 | S100A9 | RAET1G | RAET1G | TNFRSF4 | TGFB2 | TGFB2 | TGFBR2 |
| TMEM173 | TNFSF10 | TNFRSF10B | PINX1 | SMC1B | SMC2 | OMD | PDPK1 | PKN2 | TESK2 | RPA1 | SORBS1 | RGS1 | SERPINB9 | TNFSF10 | TGFBR2 | TGFBR2 | THPO |
| TNFRSF10B | TNFSF9 | TNFRSF25 | RNF8 | SMC3 | SMC3 | PTK2B | SORBS1 | PLEC | TPM1 | S100A9 | TPM1 | TLR10 | TLR10 | TNFSF15 | TNFSF8 | TNFSF9 | TNFSF9 |
| TNFSF10 | TP53BP2 | TNFSF10 | RPA1 | TGFB2 | SMC4 | S1PR1 | TENC1 | SORBS1 | TSSK2 | SORBS1 | TUBA4B | TNFSF10 | TNFSF10 | ULBP1 | TNFSF9 | UBE2L3 | UBE2L3 |
| TREX1 | TP63 | TREX1 | RPS6KA1 | TGFB3 | STAG1 | SORBS1 | TPPP | TNS1 | UBXN6 | TPM1 | UNC119 | ULBP2 | TNFSF9 | ULBP3 | UBE2L3 | UCHL1 | UCHL1 |
| YARS | TREX1 | VHL | SMC3 | USP2 | STAG2 | TPPP | VCAM1 | TPPP | UNC119 | UNC119 | WIPF1 | ULBP3 | ULBP3 | VCAM1 | UCHL1 | DBH | ZEB1 |

Abbreviations: Apop, Apoptosis; CCycle, Cell Cycle; CellAdh, Cell Adhesion; Cytosk, Cytoskeleton, ImmRes, Immune Response; Prolif, Cell Proliferation.

**Supplementary Table 2 Prediction accuracy and recall rate for the gene signatures derived from germline mutations of breast cancer tumors**

| **Dataset** | **Number of samples** | **Cancer Hallmark** | **Low-risk** | | **High-risk** | |
| --- | --- | --- | --- | --- | --- | --- |
|  |  |  | **Accuracy (%)*** | **Recall (%)^†^** | **Accuracy (%)**** | **Recall (%)^††^** |
| Training | 200 | Apoptosis 1 | 94.8 | 83.3 | 24.0 | 53.5 |
|  |  | Apoptosis 2 | 94.9 | 83.3 | 24.5 | 54.7 |
|  |  | Apoptosis 3 | 91.7 | 73.3 | 21.2 | 51.8 |
|  |  | Cell Cycle 1 | 92.3 | 76.7 | 21.1 | 49.4 |
|  |  | Cell Cycle 2 | 91.4 | 70.0 | 21.9 | 55.9 |
|  |  | Cell Cycle 3 | 91.1 | 70.0 | 21.2 | 54.1 |
|  |  | Cell Adhesion 1 | 90.1 | 66.7 | 20.2 | 53.5 |
|  |  | Cell Adhesion 2 | 84.4 | 53.3 | 14.6 | 44.7 |
|  |  | Cell Adhesion 3 | 93.7 | 80.0 | 22.9 | 52.4 |
|  |  | Cytoskeleton 1 | 86.7 | 56.7 | 16.7 | 50.0 |
|  |  | Cytoskeleton 2 | 77.0 | 23.3 | 7.0 | 45.3 |
|  |  | Cytoskeleton 3 | 78.7 | 36.7 | 9.9 | 41.2 |
|  |  | Immune Response 1 | 90.5 | 70.0 | 20.0 | 50.6 |
|  |  | Immune Response 2 | 87.9 | 60.0 | 17.8 | 51.2 |
|  |  | Immune Response 3 | 86.9 | 56.7 | 16.8 | 50.6 |
|  |  | Cell Proliferation 1 | 85.6 | 50.0 | 15.6 | 52.4 |
|  |  | Cell Proliferation 2 | 86.4 | 53.3 | 16.5 | 52.4 |
|  |  | Cell Proliferation 3 | 93.6 | 80.0 | 22.6 | 51.8 |
| TCGA-Nature | 200 | Apoptosis 1 | 89.4 | 42.2 | 9.6 | 42.2 |
|  |  | Apoptosis 2 | 93.8 | 50.6 | 13.6 | 70.0 |
|  |  | Apoptosis 3 | 88.8 | 48.3 | 8.8 | 45.0 |
|  |  | Cell Cycle 1 | 89.0 | 49.4 | 9.0 | 45.0 |
|  |  | Cell Cycle 2 | 90.6 | 48.3 | 10.6 | 55.0 |
|  |  | Cell Cycle 3 | 91.8 | 50.0 | 11.8 | 60.0 |
|  |  | Cell Adhesion 1 | 88.0 | 48.9 | 8.0 | 40.0 |
|  |  | Cell Adhesion 2 | 91.1 | 56.7 | 11.4 | 50.0 |
|  |  | Cell Adhesion 3 | 83.2 | 43.9 | 3.8 | 20.0 |
|  |  | Cytoskeleton 1 | 87.1 | 41.1 | 7.8 | 45.0 |
|  |  | Cytoskeleton 2 | 88.3 | 46.1 | 8.5 | 45.0 |
|  |  | Cytoskeleton 3 | 85.9 | 43.9 | 6.5 | 35.0 |
|  |  | Immune Response 1 | 90.4 | 41.7 | 10.3 | 60.0 |
|  |  | Immune Response 2 | 88.2 | 37.2 | 8.9 | 55.0 |
|  |  | Immune Response 3 | 88.3 | 37.8 | 8.9 | 55.0 |
|  |  | Cell Proliferation 1 | 87.0 | 44.4 | 7.4 | 40.0 |
|  |  | Cell Proliferation 2 | 93.1 | 52.2 | 13.1 | 65.0 |
|  |  | Cell Proliferation 3 | 92.2 | 52.8 | 12.4 | 60.0 |
| TCGA-CPTAC | 295 | Apoptosis 1 | 91.1 | 58.6 | 15.0 | 55.9 |
|  |  | Apoptosis 2 | 92.7 | 58.6 | 16.9 | 64.7 |
|  |  | Apoptosis 3 | 92.0 | 57.1 | 15.8 | 61.8 |
|  |  | Cell Cycle 1 | 90.1 | 52.5 | 13.3 | 55.9 |
|  |  | Cell Cycle 2 | 90.3 | 49.8 | 13.3 | 58.8 |
|  |  | Cell Cycle 3 | 89.2 | 57.1 | 12.5 | 47.1 |
|  |  | Cell Adhesion 1 | 87.2 | 49.4 | 10.2 | 44.1 |
|  |  | Cell Adhesion 2 | 86.5 | 51.3 | 9.3 | 38.2 |
|  |  | Cell Adhesion 3 | 85.9 | 53.6 | 8.3 | 32.4 |
|  |  | Cytoskeleton 1 | 87.9 | 58.6 | 10.7 | 38.2 |
|  |  | Cytoskeleton 2 | 91.1 | 54.8 | 14.5 | 58.8 |
|  |  | Cytoskeleton 3 | 91.4 | 57.1 | 15.2 | 58.8 |
|  |  | Immune Response 1 | 90.8 | 60.5 | 14.9 | 52.9 |
|  |  | Immune Response 2 | 91.4 | 60.9 | 15.7 | 55.9 |
|  |  | Immune Response 3 | 92.4 | 60.5 | 16.9 | 61.8 |
|  |  | Cell Proliferation 1 | 88.7 | 54.0 | 11.8 | 47.1 |
|  |  | Cell Proliferation 2 | 88.8 | 48.7 | 11.8 | 52.9 |
|  |  | Cell Proliferation 3 | 89.5 | 55.7 | 12.8 | 50.0 |

Notes:

*Percentage of non-recurred (i.e., non-metastatic) samples in the predicted low-risk group.

†Percentage of the predicted low-risk samples from the non-recurred group.

**Percentage of recurred (i.e., metastatic) samples in the predicted high-risk group.

††Percentage of the predicted high-risk samples from the recurred group.

**Supplementary Table 3 Constructing germline-based NOG_CSS for ER+ breast cancer by combining the training set and the testing set**

| **Number of**  **Signatures** | **Low-risk** | | **High-risk** | |
| --- | --- | --- | --- | --- |
|  | **Accuracy (%)^*^** | **Recall (%)^†^** | **Accuracy (%)^**^** | **Recall (%)^††^** |
| **1** | 84.94 | 99.54 | 15.17 | 100.00 |
| **2** | 84.88 | 99.09 | 15.29 | 100.00 |
| **3** | 85.08 | 95.47 | 15.66 | 100.00 |
| **4** | 85.65 | 91.85 | 16.03 | 97.43 |
| **5** | 87.11 | 88.68 | 17.27 | 97.43 |
| **6** | 87.20 | 83.25 | 17.96 | 94.87 |
| **7** | 89.06 | 77.37 | 18.51 | 89.74 |
| **8** | 91.27 | 71.04 | 20.46 | 89.74 |
| **9** | 92.41 | 60.63 | 23.44 | 87.17 |
| **10** | 95.65 | 49.77 | 24.34 | 71.79 |
| **11** | 95.50 | 38.46 | 27.27 | 61.53 |
| **12** | 94.36 | 30.31 | 26.47 | 46.15 |
| **13** | 96.29 | 23.5 | 24.48 | 30.76 |
| **14** | 97.50 | 17.64 | 28.57 | 25.64 |
| **15** | 100.00 | 9.95 | 21.73 | 12.82 |
| **16** | 100.00 | 4.97 | 16.66 | 5.12 |
| **17** | 100.00 | 2.26 | 0.00 | 0.00 |
| **18** | 100.00 | 1.35 | 0.00 | 0.00 |

*Notes*:

*Percentage of non-recurred (i.e., non-metastatic) samples in the predicted low-risk group.

†Percentage of the predicted low-risk samples from the non-recurred group.

**Percentage of recur (i.e., metastatic) samples in the predicted high-risk group.

††Percentage of the predicted high-risk samples from the recurred group.

Here pooling means that half of the non-recurred samples in the original training set were randomly replaced by the non-recurred samples from the validation set.

**Supplementary Table 4 Cox proportional hazards regression models of uni- and multiple-factors for breast cancer**

| **Variable** | **P-Value** | **HR** | **95% CI** |
| --- | --- | --- | --- |
| Age | 0.13 | 1.006762 | 0.998-1.016 |
| Subtype, Luminal A v Luminal B | 0.04 | 1.32708 | 1.0194-1.728 |
| Subtype, Luminal A v Unknown | 0.82 | 0.95363 | 0.6335-1.436 |
| Subtype, Luminal B v Unknown | 0.15 | 1.3874 | 0.8933-2.155 |
| Localization, Left v Right | 0.23 | 0.9035 | 0.7655-1.066 |
| Stage, I v II | 0.07 | 1.2275 | 0.98365-1.532 |
| Stage, I v III | 0.03 | 1.3261 | 1.02406-1.717 |
| Stage, I v IV | 0.13 | 0.3398 | 0.08382-1.377 |
| Stage, I v X | 0.66 | 1.2908 | 0.40870-4.076 |
| Nodal status, 0 v 1 | 0.80 | 0.9768 | 0.8134-1.173 |
| Nodal status, 0 v 2 | 0.68 | 1.0617 | 0.8020-1.406 |
| Nodal status, 0 v 3 | 0.03 | 1.4367 | 1.0440-1.977 |
| Nodal status, 0 v X | 0.54 | 0.6981 | 0.2233-2.182 |
| Subtype + Localization + Stage, Luminal A v Luminal B | 0.02 | 1.38377 | 1.05771-1.810 |
| Subtype + Localization + Stage, I v IV | 0.04 | 0.23098 | 0.05586-0.9551 |

Abbreviations: HR, hazard ratio; CI, Confidence Interval

**Supplementary Table 5 Pathway enrichment analysis of network operational signature genes from germline mutations of breast cancer patients**

| **Category** | **Term** | **FDR PValue** |
| --- | --- | --- |
| GOTERM | Antigen processing and presentation | 8.38E-09 |
| KEYWORDS | Mitosis | 2.12E-08 |
| PATHWAY | Cytokine-cytokine receptor interaction | 3.01E-08 |
| GOTERM | Cell division | 1.99E-06 |
| GOTERM | Natural killer cell lectin-like receptor binding | 2.14E-06 |
| PATHWAY | Leishmaniasis | 7.55E-06 |
| GOTERM | T cell costimulation | 1.10E-05 |
| PATHWAY | Graft-versus-host disease | 1.43E-05 |
| GOTERM | Natural killer cell mediated cytotoxicity | 1.64E-05 |
| PATHWAY | Rheumatoid arthritis | 1.74E-05 |
| PATHWAY | Inflammatory bowel disease (IBD) | 1.84E-05 |
| GOTERM | MHC class II protein complex | 4.02E-05 |
| PATHWAY | Viral myocarditis | 4.40E-05 |
| PATHWAY | Allograft rejection | 4.87E-05 |
| PATHWAY | Intestinal immune network for IgA production | 5.15E-05 |
| PATHWAY | Negative regulation of extrinsic apoptotic signaling pathway via death domain receptors | 9.50E-05 |
| PATHWAY | Antigen processing and presentation | 1.60E-04 |
| GOTERM | Mitotic chromosome condensation | 1.62E-04 |
| PATHWAY | Type I diabetes mellitus | 1.82E-04 |
| PATHWAY | Staphylococcus aureus infection | 2.39E-04 |
| PATHWAY | Influenza A | 3.50E-04 |
| PATHWAY | Toxoplasmosis | 9.54E-04 |
| PATHWAY | Asthma | 0.00131511 |
| PATHWAY | Autoimmune thyroid disease | 0.00153788 |
| PATHWAY | Tuberculosis | 0.00210254 |
| KEYWORDS | Host-virus interaction | 0.0025258 |
| PATHWAY | HTLV-I infection | 0.00252675 |
| PATHWAY | FoxO signaling pathway | 0.00487651 |
| PATHWAY | Cell cycle | 0.00936092 |
| PATHWAY | Stimulatory C-type lectin receptor signaling pathway | 0.01846333 |
| PATHWAY | Negative regulation of canonical Wnt signaling pathway | 0.03468057 |
| KEYWORDS | Host cell receptor for virus entry | 0.03776077 |
| PATHWAY | Interferon-gamma-mediated signaling pathway | 0.04199796 |
| GOTERM | Positive regulation of inflammatory response | 0.05155117 |

**Supplementary Table 6 List of immune genes abbreviations**

| **Immune gene** | **Abbreviation** |
| --- | --- |
| Activated B cells | B Cells+ |
| Activated CD4 T cells | CD4 T cells+ |
| Activated CD8 T cells | CD8 T cells+ |
| CD56bright natural killer cells | CD56bright NK cells |
| CD56dim natural killer cells | CD56dim NK cells |
| Central memory CD4 T cells | C-Memory CD4 T cells |
| Central memory CD8 T cells | C-Memory CD8 T cells |
| Effector memory CD8 T cells | E-Memory CD8 T cells |
| Effector memory CD4 T cells | E-Memory CD4 T cells |
| Eosinophils | Eos |
| Gamma delta T cells | γδ T cells |
| Immature B cells | Immature B cells |
| Immature dendritic cells | Immature DCs |
| Myeloid-derived suppressor cells | MDSCs |
| Macrophages | MΦs |
| Mast cells | MCs |
| Monocytes | Monos |
| Memory B cells | Memory B cells |
| Natural killer T cells | NK T cells |
| Natural killer cells | NK cells |
| Neutrophils | PMNs |
| Plasmacytoid dendritic cells | PC DCs |
| Regulatory T cells | Tregs |
| T follicular helper cells | Tfh cells |
| Type 1 T helper cells | T1-Th cells |
| Type 17 T helper cells | T17-Th cells |
| Type 2 T helper cells | T2-Th cells |

**Supplementary Table 7 List of immune cells abbreviations**

| **Immune cell** | **Abbreviation** |
| --- | --- |
| Activated Natural killer cells | NK cells+ |
| Activated dendritic cells | DCs+ |
| Activated mast cells | MCs+ |
| Activated memory CD4 T cells | CD4 memory T cells+ |
| Eosinophil | Eos |
| Gamma delta T cells | γδ T cells |
| Macrophage M0 | M0-MΦs |
| Macrophage M1 | M1-MΦs |
| Macrophage M2 | M2-MΦs |
| Monocyte | Monos |
| Naive B cells | Naïve B cells |
| Naive CD4 T cells | CD4 naïve T cells |
| Neutrophil | PMNs |
| Plasma cells | PCs |
| Regulatory T cells | Tregs |
| Resting Dendritic cells | DCs- |
| Resting Natural killer cells | NK cells- |
| Resting mast cells | MCs- |
| Resting memory CD4 T cells | CD4 memory T cells- |
| T follicular helper cells | Tfh cells |

**Supplementary Table 8 Sample filtering steps in breast cancer dataset**

| **Dataset** | **Clinical Information** | **Sequencing** | **Training Set** | **Testing Set** | **Validation Set 1 (TCGA-CPTAC)** | **Validation Set 2 (TCGA-Nature)** |
| --- | --- | --- | --- | --- | --- | --- |
| Breast | 1067 | 755 | 200 | 60 | 295 | 200 |

**Supplementary Methods**

**Sequencing data pre-processing and variant calling**

The GATK^1^ pipeline-based whole exome-sequence data pre-processing was described previously^2^. Briefly, duplicate reads were marked and removed using GATK’s markDuplicates removed using BamTools^3^. Reads with a low mapping quality (n=60) were also removed using BamTools. Local realignment around indels was made using GATK’s IndelRealign/RealignTargetCreator and finally, base recalibration was conducted using GATK’s BaseRecalibrator. All the variants were obtained using the Varscan2^4^ somatic option by analyzing normal/tumor matched sequencing files. Variants with strand-specific bias, coverage less than 30 reads and variant frequency for heterozygous calls of less than 0.08 were removed.

**Determine tumor purity**

Tumor purity was obtained using absCNseq^5^. To run absCNseq, for a given tumor, we generated a segmentation file and a SNV (Single Nucleotide Variants) file. The segmentation file was generated by running Varscan2 using the standard protocol. Briefly, we ran VarScan2’s copyNumber on normal and tumor BAM files, and VarScan2’s copyCaller to adjust for GC content and finally applied circular binary segmentation. The SNV file was then transformed from the VCF file by running VarScan2. For some samples, absCNseq could give a few purity solutions. In this situation, the consensus purity was selected. The samples with purity greater than 70% were retained for downstream analyses. Ultimately, 755 ER+ breast tumor samples were available for further analysis.

**Training and validation sets for ER+ breast cancer dataset**

To identify gene signatures of ER+ breast cancer, we randomly selected 200 samples, which have follow-up time, as the training set (30 and 170 for recurred and non-recurred samples, respectively). By default, ~15% of ER+ breast tumors get recurred within 10 years^6^. Clinically, at present almost all of the ER+/luminal breast cancer patients receive tamoxifen treatment. However, tamoxifen treatment for the ‘real’ low-risk patients does not affect patients’ survival. To develop gene signatures for prognosis, we controlled the training set such that we tried to make sure that the selected ‘low-risk’ patients are ‘real low-risk’ patients by applying these rules: (1) the low-risk patients who have relatively longer survival in the cohort, (2) we further confirmed them by predicting them using the gene-expression-based prognostic signatures we developed previously^7^. This signature was developed using a cohort where the patients have not been treated with any chemotherapy. The predicting accuracy for low-risk ER+ breast cancer reached 95%. Except these training samples, the rest of the ER+ breast tumor samples in the GDC was used for validation. Sixty non-recurred samples were retained for obtaining optimal signature cutoffs (Table S8). Finally, all the remaining ER+ samples were separated into 2 validation sets (TCGA-Nature, TCGA-CPTAC) composed of 200 and 295 samples, respectively. For TCGA-Nature set, we used a ratio of 10% of recurred samples (20 recurred and 180 non-recurred samples). For TCGA-CPTAC, we used a ratio of 11.5% of recurred samples (34 recurred and 261 non-recurred samples).

**Oncotype Formula Calculations**

The recurrence score (RS) for all samples for tumoral RNA-seq was calculated based on previous publications^8-10^. Proteins were transformed into genes:

HER2 Group Score (0.9 x *GRB7*) + (0.1 x *ERBB2*)

If HER2 Group Score is less than 8 then the HER2 Group Score is considered equal to 8

# ER Group Score ([0.8 x *ESR1*] + [1.2 x *PGR*] + *BCL2* + *SCUBE2*)/4

Proliferation Group Score (*BIRC5* + *MKI67* + *MYBL2* + *CCNB1* + *AURKA*)/5

If the Proliferation Group Score is under 6.5 then the Proliferation Group Score is considered equal to 6.5

Invation Group Score (*CTSV* + *MMP11*)/2

RS_U_ = + 0.47 x HER2 Group Thresholded Score

- 0.34 x ER Group Score

+ 1.04 x Proliferation Group Thresholded Score

+ 0.10 x Invasion Group Score

+ 0.05 x *CD68*

- 0.08 x *GSTM1*

- 0.07 x *BAG1*

Cutoffs for groups were initially set as RS < 18 for low-risk, RS >= 37 for high-risk and RS between 18 and 37 for intermediate-risk with a RS scale between 0 and 100. Because our RS scale was different than the ones obtained for RT-qPCR, we had to adjust these cutoffs accordingly. Samples were sorted based on their RS. Bottom 17% of samples were assigned to the low-risk group (lowest RS), top 37% of samples were assigned to the high-risk group (highest RS) and finally, all other samples were assigned to the intermediate-risk group.

**Leukocyte mutations comparative analysis**

To further assess leukocyte variants predictive power, we re-ran eTumorMetastasis^11^ pipeline by using only germline functional variants located in leukocyte metagenes^12^. The immune metagenes list contained 785 genes. 460 of them were included in the ER+ breast cancer recurrence specific network and were retained for this analysis. We used the same training and validation sets as well as the same cutoffs used previously but using functional germline immune metagenes variants as seeds for network propagation for each sample. Gene sets were generated randomly for each GO Terms (Apoptosis, Cell Cycle, Cell Adhesion, Cell Proliferation, Immune Response and Cell Proliferation) and then we applied the MSS algorithm^7,11^. For each GO Term, not enough significant gene sets were obtained for us to be able to extract a NOG gene signature. For each sample, the number of seeds was very small due to our starting pool 460 immune genes. Such a small number of seeds in the network resulted in a much weaker signal which was not sufficient to distinguish recurred and non-recurred groups. Using the whole germline landscape of patients provides a lot more functional variants which generate a stronger signal. This also suggests that many other germline variants might act as regulators to immune genes affecting tumor recurrence.

**Random network comparative analysis**

To further examine the importance of generating an ER+ breast cancer specific recurrence network in eTumorMetastasis pipeline, we generated a random network to replace the ER+ breast cancer specific recurrence network. To do so, we retained all the genes in our ER+ breast cancer network but assigned them random interactions. The same number of genes (n=6184) and interactions (n=62004) were used in the random network than those in the breast cancer specific recurrence network. By comparison, 184 interactions (0.03%) were shared between both networks, suggesting that the random network was very different from the breast cancer specific recurrence network. Then, we re-ran network propagation using the same seeds previously found in each sample. Finally, we ran a simple student t-test with the new propagation scores between both groups (low- and high-risk) and found only 287 significantly modulated genes. For comparison, 712 genes were found when using the breast cancer specific recurrence network. These results highlight that breast cancer specific recurrence network is a context-dependent network representing the breast cancer recurrence which is a critical component for biomarker identification.

**Supplementary References**

1 DePristo, M. A. *et al.* A framework for variation discovery and genotyping using next-generation DNA sequencing data. *Nat Genet* **43**, 491-498 (2011).

2 Zaman, N. *et al.* Signaling network assessment of mutations and copy number variations predict breast cancer subtype-specific drug targets. *Cell Rep* **5**, 216-223 (2013).

3 Barnett, D. W., Garrison, E. K., Quinlan, A. R., Stromberg, M. P. & Marth, G. T. BamTools: a C++ API and toolkit for analyzing and managing BAM files. *Bioinformatics* **27**, 1691-1692 (2011).

4 Koboldt, D. C. *et al.* VarScan 2: somatic mutation and copy number alteration discovery in cancer by exome sequencing. *Genome Res* **22**, 568-576 (2012).

5 Bao, L., Pu, M. & Messer, K. AbsCN-seq: a statistical method to estimate tumor purity, ploidy and absolute copy numbers from next-generation sequencing data. *Bioinformatics* **30**, 1056-1063 (2014).

6 Voduc, K. D. *et al.* Breast cancer subtypes and the risk of local and regional relapse. *J Clin Oncol* **28**, 1684-1691 (2010).

7 Li, J. *et al.* Identification of high-quality cancer prognostic markers and metastasis network modules. *Nat Commun* **1**, 34 (2010).

8 Paik, S. *et al*. A multigene assay to predict recurrence of tamoxifen-treated, node-negative breast cancer. *N Engl J Med* **351**, 2817-26 (2004).

9 Paik, S. *et al*. Gene expression and benefit of chemotherapy in women with node-negative, estrogen receptor-positive breast cancer. *J Clin Oncol* **24**, 3726-34 (2006).

10 Sinicropi, D. *et al*. Whole Transcriptome RNA-Seq Analysis of Breast Cancer Recurrence Risk Using Formalin-Fixed Paraffin-Embedded Tumor Tissue. *PLoS One* **7**, e40092 (2012).

11 Milanese, J.S. *et al*. eTumorMetastasis, a network-based algorithm predicts clinical outcomes using whole-exome sequencing data of cancer patients. Preprint at: https://doi.org/10.1101/268680 (2018).

12 Charoentong, P. *et al*. Pan-cancer Immunogenomic Analyses Reveal Genotype- Immunophenotype Relationships and Predictors of Response to Checkpoint Blockade. *Cell Rep*. **18**, 248-262 (2017).
